# Asthma in Children and Adults—What Are the Differences and What Can They Tell us About Asthma?

**DOI:** 10.3389/fped.2019.00256

**Published:** 2019-06-25

**Authors:** Michelle Trivedi, Eve Denton

**Affiliations:** ^1^Division of Pediatric Pulmonology, Department of Pediatrics, University of Massachusetts Medical School, Worcester, MA, United States; ^2^Department of Population and Quantitative Health Sciences, University of Massachusetts Medical School, Worcester, MA, United States; ^3^Department of Respiratory Medicine, Alfred Hospital, Melbourne, VIC, Australia; ^4^Public Health and Preventive Medicine, Monash University, Melbourne, VIC, Australia

**Keywords:** asthma, pediatric, adult, childhood, airway

## Abstract

Asthma varies considerably across the life course. Childhood asthma is known for its overall high prevalence with a male predominance prior to puberty, common remission, and rare mortality. Adult asthma is known for its female predominance, uncommon remission, and unusual mortality. Both childhood and adult asthma have variable presentations, which are described herein. Childhood asthma severity is associated with duration of asthma symptoms, medication use, lung function, low socioeconomic status, racial/ethnic minorities, and a neutrophilic phenotype. Adult asthma severity is associated with increased IgE, elevated FeNO, eosinophilia, obesity, smoking, and low socioeconomic status. Adult onset disease is associated with more respiratory symptoms and asthma medication use despite higher prebronchodilator FEV1/FVC. There is less quiescent disease in adult onset asthma and it appears to be less stable than childhood-onset disease with more relapses and less remissions.

## Childhood Onset Asthma

### Introduction to Childhood Asthma

Childhood asthma is not a singular disease, but rather a uniquely diverse disorder with variable presentation throughout childhood. Asthma affects 8.3% of children in the United States and is the most common chronic disease of childhood (1, 2). Childhood asthma is responsible for 50 billion dollars in annual healthcare expenditures and is a major cause of emergency room visits, hospital admissions, school absences, and loss of parental workdays (1–3).

Asthma is characterized by inflammation leading to bronchoconstriction, edema, and increased mucous production in the airways. Interestingly, the disorder is more prevalent in boys in the first decade of life. However, after puberty and in the second decade of life, it appears that asthma is more prevalent in young women (4). Asthma disproportionately affects minority and low-income children with African American and Hispanic children having the highest prevalence rates, morbidity and mortality due to asthma (5, 6).

Asthma is considered a chronic disease of childhood however there are periods of time during which disease can go into remission or resolve altogether. Important risk factors for the development of childhood asthma have been identified. The phenotypes of childhood asthma and varied presentations are best defined through the periods of the pediatric life course and are described herein.

### Childhood Asthma Risk Factors

The perinatal period has been implicated in the development of childhood asthma. Several cohort studies have unveiled risk factors for the development of asthma in offspring, with factors that span from genetic and environmental risk factors to features such as child's sex and presence of atopy.

#### Genetic Risk Factors

The genetics of asthma are an emerging and complicated topic. Multiple genes are thought to contribute to asthma and rapidly changing technology continues to build our current understanding of the genetic risk factors for asthma development. This is a complex topic that we will only briefly describe herein. Genome-wide Association Studies (GWAS) have dramatically improved our understanding of asthma susceptibility genes. Briefly, the following genes have been determined to have significant association with asthma susceptibility: the 17q21 locus with the *ORMDL3 and GSDML genes, the IL33 gene* on chromosome 9p24, the *HLA- DR/DQ gene* on chromosome 6p21, the *IL1RL1/IL18R1gene* on chromosome 2q12, the *WDR36/ TSLP gene* on chromosome 5q22 and the *IL13gene* on chromosome 5q31 (7). Interestingly, GWAS have shown evidence that loci may be specific to racial/ethnic populations, such PHYNN1 observed in African-Americans with asthma (8). However, as with other common diseases, for individuals, only a small degree of heritability of asthma can be explained by the genes observed in GWAS. Therefore, emerging and multi-genetic approaches are needed to further study the genetic susceptibility to asthma (7).

#### Environmental Risk Factors

Environmental perinatal risk factors are also important to consider for childhood asthma. Maternal tobacco smoking during pregnancy has been shown to increase the risk of childhood asthma (9). Maternal diet in pregnancy has also been implicated as an asthma risk factor with reports of maternal diets higher in vitamin E, zinc, and polyunsaturated fatty acids as protective against the development of childhood asthma (10–12). In contrast, high sugar intake in the maternal diet during pregnancy has been associated with increased risk of asthma in offspring (13). Other maternal dietary factors have been studied but with less conclusive results including the intake of vitamin D, vitamin C, and a Mediterranean diet. Other perinatal risk factors for childhood asthma that have been reported are neonatal jaundice, maternal preeclampsia, and cesarean section delivery, all which have been associated with higher risk of childhood asthma development (14–16).

Ultimately gene-environment interactions (the genetic-environmental axis) are critical for the development of asthma in a child (8).

#### Natal Risk Factors

Chronic lung disease of prematurity is known to increase the risk of asthma development in children (17). Specifically, extreme preterm birth (23–27 weeks gestation) is associated with an increased risk of asthma into young adulthood (18). Additionally, cesarean section delivery as mode of delivery (16, 19) and low birth weight have been associated with asthma diagnosis in mid-childhood with symptoms persisting into adult life (20).

#### Sex

Boys are more likely to develop childhood asthma, as compared with girls, at least until the point of puberty. This has been explained by smaller airway size in boys compared with girls under age 10 years, which predisposes to worsened airway reactivity, as compared with girls of the same age, height and weight (21).

#### Family History

Both maternal and paternal histories of asthma are associated with increased risk of asthma in offspring. Interestingly, maternal asthma history is more strongly associated with asthma development in the child (22).

#### Medical History

Presence of atopy (having IgE antibodies to specific allergens) is strongly associated with childhood asthma (23). Specifically the “atopic march” is a pattern that is described clinically in individuals with atopic disease. This “atopic march” begins as atopic dermatitis (or eczema) in infancy, develops on to allergic rhinitis (or hayfever) and then asthma later in childhood (24). Specific indoor allergen sensitization in early life have been of interest with regard to asthma development. Sensitization to house dust mite, alternaria mold, and cockroach allergens have been associated with increased risk of asthma (25, 26), whereas early life exposure to cat and dog allergens have been associated with both increased and decreased risk of asthma in different studies (27, 28).

#### Medication Exposure

Exposures to antibiotics (29) and antipyretics (30) in infancy have been described to be associated with increased risk of developing childhood asthma however the data has been conflicting and the study results have been concerning for uncontrolled confounding bias. Therefore, further studies are warranted before conclusions can be made about these associations.

### Presentation of Asthma: Early Childhood (0–6 Years)

Studies of asthma's natural history have shown that almost 80% of cases begin during the first 6 years of life (31). The symptoms of pediatric asthma in this age group are varied and not specific to asthma making the diagnosis challenging. The primary symptoms of asthma in infancy and early childhood include cough, both dry and productive (albeit young children rarely expectorate), wheeze, shortness of breath, and work of breathing. Asthma symptoms are a result of airway inflammation, bronchospasm, airway edema, and airway mucous gland hypertrophy. Interestingly, these symptoms can also present with a multitude of other pediatric diseases including respiratory tract infections and congenital airway anomalies posing a diagnostic challenge. It is well-established that asthma in this age group is frequently under-diagnosed and undertreated (32, 33).

Often, clinicians including pediatric asthma specialists (pulmonologists and allergists) define asthma in this age group as symptoms of airway inflammation that reverse with bronchodilator therapy. However, given the diagnostic challenge in this age group, the Asthma Predictive Index (API) was developed to guide the diagnosis of childhood asthma in children under age 3 years (34). The API has limited sensitivity but reasonable specificity. The API major criteria were defined as physician-diagnosed eczema or parental asthma. The minor criteria were defined as physician-diagnosed allergic rhinitis, wheezing apart from colds, and serum eosinophilia >4%. Positive loose index were defined as parental report of wheezing on surveys at age 2 or 3 years and either 1 major or 2 minor criteria. Positive stringent index was defined as frequent wheezing on surveys at age 2 or 3 years and either 1 major or 2 minor criteria. Children with a positive loose index were found to be four times more likely to have active asthma on surveys at age 6, 8, 11, and 13 years (sensitivity 42%, specificity 85%). Children with a positive stringent index were seven times more likely to have asthma on a school-age survey (sensitivity 16%, specificity 97%). The API is most useful for its negative predictive value and thus, when negative, is an essential tool for determining who is not likely to go on to having later asthma. While not a perfect tool, a slightly modified API, with criteria of a higher score of frequent wheezing and replacing “physician-diagnosed allergic rhinitis” with skin prick testing, is endorsed by the US National Asthma Education and Prevention Program Expert Panel Report 3 for use in diagnosing asthma in this young age group (35).

Often in this age group, particularly over 0–3 years, symptoms are virally triggered rather than allergically triggered. Infants will often have very few symptoms until they experience an upper respiratory infection, which can trigger a significant and severe inflammatory cascade.

In children, the initial few years following asthma diagnosis are critical. For both physician visits and hospitalizations, the number of children having had a second asthma encounter peaked at 3 years after diagnosis and then stabilized (36). Overall, 75% of children had a second asthma episode within 3 years of diagnosis, suggesting that it takes ~3 years to control and stabilize asthma episodes (36). The frequency of asthma episodes soon after diagnosis points to the need for attentive follow-up and aggressive management and education strategies in the early years (36). The mainstays of therapy in this age group are based on recurrence of wheezing symptoms or in severity. For those children with recurrent wheezing or significant morbidity with multiple emergent visits, oral steroid courses or hospital admissions, inhaled corticosteroids are the main therapy. There are a limited number of pathophysiological asthma studies in children under 5 years of age, which presents a challenge in the evidence base for the management of childhood asthma.

### Presentation of Asthma: Late Childhood (7–11 Years)

By this age, children can more reliably perform spirometry, and reversible airway obstruction on spirometry can be a helpful diagnostic tool. However, it is important to note that in children with asthma, spirometry values can be normal despite significant disease and morbidity (37). Therefore, in children, spirometry is often used as a monitoring tool for asthma symptoms after the diagnosis has been established through other assessments (38).

Symptoms in this age group transition more from discrete episodes of wheezing in response to viral infections to allergic triggered exacerbations. In this age group, exercise-induced symptoms manifest more clearly which may be due to a true change in the clinical presentation of asthma in this age group or also due to sports and exercise becoming a more discreet activity for children of this age wherein caretakers are able to appreciate the symptoms of dyspnea or cough with exertion. In children who avoid or develop a loss of interest in exercise or physical activities, it is important to consider that asthma may be underlying.

Some children in this age group will have few day-to-day symptoms, but have severe asthma attacks in response to specific triggers such as cold weather, cigarette smoke, or seasonal allergies. Virally triggered asthma exacerbations occur in this age group but less often than in the 0–6 year age range and may contribute to the lower rates of healthcare utilization in this age group as compared with younger years of 0–4 years (2).

### Presentation of Asthma: Adolescence (12–18 Years)

Puberty has an interesting impact on childhood asthma, specifically relating to sex. Prior to puberty, asthma risk is higher among male children. At the time of puberty, the risk of asthma is approximately equal between males and females, and after puberty, girls have a higher risk of asthma (39). Some of these differences could be explained by the differences in airway development between the sexes. The fetal lung is less developed in boys from 16 to 26 weeks, measured by mouth movements that reflect fetal breathing, a critical determinant of lung development. In the last 4 weeks of gestation, airway resistance is higher in males (21). Boys up to 10 years old appear to have smaller airways in relation to lung size as compared with girls of the same age, height, and weight (21). After puberty, smaller airway caliber is then observed in the female sex (39). The known sex differences in asthma may also be due to other factors such as hormonal effects, genetic susceptibility, immunologic response, and differences in consultation practices and health-seeking behaviors by sex (4, 40).

Asthma symptoms in this age group are most predominantly shortness of breath with exertion, wheezing in response to triggers, chest pain, chest tightness, and cough. In this age group, asthma symptoms can significantly impact sleep, school, sports, and social engagements. Children are more aware of symptoms in this age range and often feel more embarrassment or stigma around using an inhaler and in particular a spacer, often leading to under treatment of asthma symptoms (40).

Remission is common in adolescence, with remission rates reported at 16–60% (41). Factors that have been implicated in an increased probability of asthma remission include mild disease and minor airway inflammation before adolescence, male sex, and the absence of allergic sensitization (42, 43).

### Wheezing and Asthma Phenotypes in Childhood

Childhood wheezing phenotypes have been explored given that nearly 50% of children experience wheezing before age 1, yet only 20% of those children progress to have continued wheezing later in childhood (44). While there are several longitudinal birth cohorts that have described wheezing phenotypes, we will describe the classifications according to the earliest of these studies: the Tucson Children's Respiratory Study and the most recent systematic comparison of the clinical and epidemiologic classifications (45).

## Wheezing Phenotypes (44, 45):

### Never/Infrequent Wheeze

This Describes Children who do not Experience any Wheezing.

### The Transient Wheeze

This describes children who have their first wheeze before the age of 3 years with resolved wheezing by age 6 years. Transient wheeze is not strongly related to atopy and genetic risk; there are only mild impairments in lung function in this phenotype, and medication use is very uncommon (45).

### The Persistent Wheeze

This describes children who experience first wheezing before age 3 year, however go on to have continued wheezing at age 6 years. Persistent wheeze was strongly related to the asthma risk locus on chromosome 17, however this phenotype appears to be unrelated to environmental determinants. Interestingly, bronchodilator administration dramatically improved any compromises in lung function for children with this phenotype (45).

### Intermediate Onset Wheeze

This describes children who experience rare (or no) wheezing before 18 months of age, but persistent wheeze thereafter. Intermediate-onset wheeze has associations with atopy, but only to pollen sensitization (45).

### The Late Onset Wheeze

These children develop wheezing between age 3 and 6 years. Late-onset wheeze is strongly associated with fractional exhaled nitric oxide levels and sensitization to inhaled allergens at 6 years and at 4 years. There appears to be severe and irreversible reduction in lung function in this phenotype and asthma medication use is common (45).

## Childhood Asthma Clinical Phenotypes

### Asthma Diagnosis (45)

Physician's diagnosis of asthma at least once per lifetime or recurrent diagnoses of spastic, obstructive, or asthmatic bronchitis as reported by the parents at age 6 years.

### Frequent Wheeze (45)

Wheeze on a monthly basis for at least 1 year between age 1 and 6 years.

### Unremitting Wheeze (45)

Having symptoms between wheezing episodes or having wheeze without a cold at least once between age 1 and 6 years.

### Recurrent Unremitting Wheeze (45)

Having *s*ymptoms between wheezing episodes or wheeze without a cold for 2 or more years between age 1 and 6 years.

### Multi-Trigger Wheeze (45)

Having at least 2 common asthma triggers leading to wheeze between ages 3–6 years.

### Episodic Wheeze (45)

Wheezing episodes associated only with viral upper respiratory infection between age 1 and 6 years.

### Severe, Difficult to Control Asthma, Steroid-Resistant Asthma

Children that do not seem to respond to standard treatment are referred to as severe or difficult to control asthma, and these children experience substantial morbidity from asthma symptoms. To classify a child into this phenotype, the first step is to exclude an incorrect diagnosis, poor adherence to treatment, or incorrect technique with an inhaler and spacer (46, 47). Supervised asthma therapy programs can be extremely useful in managing asthma symptoms and reducing healthcare utilization for children with poor medication adherence and inhaler and spacer technique (48, 49). It is important to differentiate between severe therapy-resistant asthma and difficult-to-treat asthma due to comorbidities Difficult to treat asthma is a much more common reason for persistent symptoms and exacerbations and can be managed if comorbidities, such as allergic rhinitis and chronic exposure to asthma triggers, are directly targeted. Home visiting programs and assessment of the school environment are important features of the evaluation for children with concern for chronic exposure to asthma triggers (50–52). Children with persistent symptoms and exacerbations despite correct inhaler technique and good medical adherence to standard asthma therapy (steroid-resistant or therapy resistant asthma) should be referred to an asthma specialist to consider more potent biologic therapies such as anti-IgE, anti-IL-5, or anti-IL-13 therapies and further evaluation (47).

### Eosinophilic Predominant

Eosinophilic inflammation is considered to be the main feature of allergic asthma in children (53). Sputum eosinophils and serum periostin are biomarkers that have been proposed for defining which children with asthma will respond to anti-IgE, anti-IL-5, or anti-IL-13 asthma treatment (54). However bronchoscopy is not routinely done in children with asthma, therefore serum eosinophils are often the least invasive and most common biomarker utilized to indicate the presence of eosinophilic predominant asthma and help predict responsiveness to inhaled corticosteroid therapy (55).

### Neutrophilic Predominant

While initially most childhood asthma was thought to be eosinophilic in nature, a neutrophilic predominance has emerged as an important phenotype. In this phenotype, children generally have low IgE levels, low serum and sputum eosinophil counts with very little allergic symptoms. Neutrophil-predominant asthma is the most severe asthma phenotype with poor corticosteroid response (54) and may explain some of the children who have not respond well to standard asthma therapy.

## Other Childhood Asthma Clinical Presentations:

In clinical practice, there are different clinical presentations of symptoms that point to an underlying diagnosis of childhood asthma, and clinical improvement can occur in response to starting a child on preventive asthma therapy, such as a daily-inhaled corticosteroid and use of bronchodilator therapy for acute episodes.

### Recurrent Croup

Croup, inflammation of the upper airway, which presents as barky cough and stridor, is a common isolated entity in infancy and early childhood. However, the presence of recurrent croup may indicate the presentation of underlying asthma in childhood. Recurrent croup has been shown to be a risk factor for childhood asthma and airway hyperreactivity (56) as well as strongly associated with bronchial asthma in children (57).

### Middle Lobe Syndrome

Asthma in children is associated with significant atelectasis and specifically with middle lobe and lingula collapse, (58) and often infants and children who ultimately develop asthma, present as recurrent atelectasis, or mucous plugging in the right middle lobe and lingula. In contrast to adults, children are thought to have higher resistance to collateral airflow, possibly due to increasing number and size of collateral alveolar ventilation, through pores of Kohn and bronchoalveolar Lambert's channels, that develop from birth to adulthood (59). This theory is supported by the finding that pores of Kohn are absent in newborns, and develop around 4 years of age, with the greatest numbers of pores of Kohn found in the apical portions of upper and lower lobes, as well as in peribronchial, perivascular, and subpleural areas, (60, 61), leaving the right middle lobe and lingual vulnerable to atelectasis and mucous plugging, particularly in children with asthma.

### Recurrent Pneumonia

In a child with recurrent multi-lobar pneumonia, with a normal immune function evaluation, asthma should be considered as an underlying cause of the recurrent chest infections. Many children referred to specialty care with recurrent chest infections will be found to have undiagnosed or undertreated asthma. Often the history reveals that most have recurrent episodes of cough, wheeze and breathlessness, with trigger factors of upper respiratory tract infections, exercise, cold air, emotional upset, or exposure to pets and other aero-allergens suggestive of asthma (62). In several studies, asthma was the main underlying cause for recurrent pneumonia in children (63).

### Association Between Childhood Asthma and COPD

Children with asthma have an increased risk of developing chronic obstructive pulmonary disease (COPD) in adulthood. Specifically, it has been shown that children who smoke tobacco and also have asthma are at increased risk for developing low lung function and COPD as adults, when compared to smokers who did not have asthma in childhood (64).

### Remission and Mortality in Childhood Asthma

Asthma remission occurs most commonly between the ages of 14–21 years (65). However, large longitudinal studies have also shown that, among children who wheezed before age 3 years, more than 50% had stopped wheezing either by 6 years of age (44) or by 12 years of age, (36, 66) depending on the study. Remission rates of childhood asthma have been reported between 16 and 60% by early adulthood, according to prior longitudinal studies (41, 65, 67). The wide variation in reported remission rates is likely due to diverse study designs, varying follow-up periods, and different study populations. In longitudinal studies, children with the following characteristics had higher remission rates: episodic asthma (rather than persistent asthma), milder initial asthma severity, less allergic sensitization, less allergic rhinitis, less atopic dermatitis, and male sex (36, 65).

While the morbidity of childhood asthma is significant, fortunately, mortality from childhood asthma is rare with an estimated 28 deaths per million children with asthma (2). As with childhood asthma morbidity, there are grave racial disparities in childhood asthma mortality, and black, and Hispanic children suffer disproportionately from the highest mortality rates (2).

## Adult-Onset Asthma

### Introduction to Adult-Onset Asthma

Asthma is increasingly recognized as an umbrella term for a heterogenous group of conditions that has been likened to the term “arthritis” in Rheumatology—not a specific diagnosis but a term that describes a diverse group of conditions clinically and biologically (68). Some have suggested discarding the term asthma altogether (69).

Asthma is a common condition amongst adults, estimated to affect 235 million people worldwide, and is estimated to cause more than 350,000 deaths per year (70). It carries a huge economic, morbidity and mortality burden in both developing and developed nations (70). The mortality in developed countries from asthma has remained static for more than a decade and it is clear that a better understanding and management of this diverse group of conditions is required (71).

Asthma is considered a childhood disease by many but this is erroneous as longitudinal studies have shown that approximately half of middle aged patients with asthma have had onset in adulthood rather than childhood (72–74). This proportion of adult onset asthma increases with age (73). The annual incidence of asthma amongst adults is estimated to be 0.5%, similar to the incidence in childhood and it remains unclear as to whether adult-onset asthma is a different disease to that occurring in childhood (75).

The area of asthma phenotyping amongst adults has been developing at a rapid rate. Initially clinical phenotypes were identified that help to categorize asthma in adults by clinical traits including that of early and late onset, obese vs. non-obese, and atopic vs. non-atopic (76, 77). More recently biologic phenotyping has been increasingly recognized and performed clinically, particularly with the availability of targeted biologic agents, and this phenotyping is currently based on presence of allergic or eosinophilic inflammation although this is a rapidly evolving field (68). This has led on to more complex molecular phenotyping which may well-inform future precision medicine in asthma (78).

The natural history of asthma occurring in adulthood is complex because it is such a heterogenous disease. Despite the complexities adult-onset asthma appears to run a different course to that of childhood-onset disease where the majority of the disease is mild and remission is common (79) (Table 1). In adults with asthma remission is uncommon and disease is often more severe and progressive (80).

**Table 1:** Comparison of childhood and adult asthma.

**Feature**	**Childhood asthma**	**Adult asthma**
Variable Phenotypes	Yes	Yes
Symptoms	Age 0–6 years: Cough, wheezing, acute episodes of dyspnea and increased work of breathing Age 6–11 years: cough, wheezing, dyspnea or cough with exertion Age 11–18 years: cough, wheezing, dyspnea, dyspnea on exertion, chest tightness, chest pain	Shortness of breath, wheeze, chest tightness and cough. *Allergic bronchopulmonary aspergillosis:* Above symptoms plus sputum production. *Aspirin exacerbated respiratory disease:* Above symptoms plus nasal polyps and sensitivity to aspirin.
Sex predominance	Males < 10 years	Females
Remission	Common	Rare
Factors associated with severity	Asthma duration, medication use, lung function, neutrophilic phenotype, low socioeconomic status, racial/ethnic minorities	IgE, FeNO, eosinophilia, obesity, smoking, low socioeconomic status
Mortality	Rare	Uncommon

### Phenotypes

Although there has been significant development and research into asthma phenotypes in the past two decades the concept has developed slowly and to some remains controversial (68). In this context phenotype refers to subtypes of the disease that have recognizable properties produced by interactions of the genotype and the environment (81). From this concept has emerged the concept of asthma endotypes where a biologic pathway is identified that leads to the clinical phenotype (82). Phenotypes and endotypes are an attractive concept but unfortunately patients rarely fit into these classifications perfectly with many factors that muddy the waters including presence of comorbidities and confounders. For this reason a more pragmatic concept of treatable traits has gained momentum (83). Rather than attempting to categorize people with asthma, this approach seeks to describe traits associated with an individual's asthma including clinical aspects, biological characteristics, and comorbidities (83).

Phenotypes of asthma can be broadly categorized into *clinical, biological*, and *molecular* although there is considerable overlap between these and, ultimately, it is the combination of the three that is likely to form the asthma phenotypes of the future.

#### Clinical

The attempt to classify asthma is not a new concept with clinical phenotypes having been described since the 1940s (84, 85). One of the oldest approaches to clinical phenotyping was between allergic (extrinsic) and non-allergic (intrinsic) asthma that described two clinically distinct entities—that of early onset asthma associated with sensitization to allergens and other allergic diseases, and that of the poorly understood late onset asthma that was non-allergic (84). Because these phenotypes did not confer specific management there was little clinical utility in the distinction.

More recently different clinical phenotypes have been identified by unbiased cluster analysis of cohorts of asthma patients, with the most detailed analysis performed on the Severe Asthma Research Program cohort in the United States (76, 77). This analysis resulted in the development of five clinical groups, two with early onset, and three other groups. The two groups with early onset are those with normal lung function requiring minimal controller medications, and those with preserved lung function but requiring more medications and healthcare utilization (77). The first of the other three groups comprises obese women with late onset non-atopic asthma, moderately impaired lung function and frequent oral corticosteroid use for asthma exacerbations. The remaining two groups comprise those with severe airflow obstruction and bronchodilator responsiveness, and a less well-defined group with variable ability to attain normal lung function, age of onset, atopic status, and use of oral steroids.

These phenotypes have been further refined with the addition of biologic markers to five groups; early-onset allergic, late-onset eosinophilic, exercise-induced, obesity-related and neutrophilic (68). These will be discussed in more detail below.

The extension of the development of more detailed clinical phenotypes is whether these phenotypes represent distinct clinical entities with different treatment strategies. There is considerable work being done currently in this space.

There are, however, a number of clinical phenotypes amongst adults with asthma that are distinct, including those with occupational asthma, aspirin-associated asthma, and asthma associated with other conditions such as allergic bronchopulmonary aspergillosis and chronic obstructive airways disease (COPD) (86–88). Some of these subtypes have more developed clinical characteristics associated with them, biological mechanisms, management, and natural history.

#### Biologic

As phenotyping of asthma has progressed there has been progressive attempts to describe the endotype, or biologic pathway, behind different clinical phenotypes.

The most developed of these is the distinction between the presence or absence of Type 2 inflammation (T2) in asthma (89). It has long been recognized that for many, but not all, patients with asthma there is upregulation of Type 2 inflammation. This is characterized by an stimulus at the level of the airway epithelium that results in production of IL-25, IL-33, and thymic stromal lymphopoietin (TSLP) that stimulate release of IL-4, IL-5, and IL-13 and, in turn eosinophils and antibodies that lead to the pathogenic airway changes characteristic of asthma (90). This upregulation of the Type 2 inflammatory response in asthma has important clinical implications because this inflammation tends to be responsive to corticosteroids.

Unlike in children, amongst adults there is a significant proportion of non-T2 high disease. This group of patients is less well-understood than their T2-high disease counterparts and accounts for a significant proportion of mild-moderate adult-onset asthma (91). Although these patients are poorly characterized there are significant implications for treatment as they tend to be less steroid responsive than those with T2-high disease and it is unclear what the best treatment strategy for these patients is (92).

Most asthma, even amongst adults, still appears to have T2-high etiology (76, 77). Even clinical subtypes such as exercise-induced asthma appears to be T2-mediated (93). With regard to the asthma phenotypes discussed above, different biologic mechanisms have been described. Early-onset allergic asthma is the predominate form in children, varies in severity, is associated with allergic symptoms and other allergic diseases and characterized by elevated specific IgE, T2 cytokines and is responsive to inhaled corticosteroids (68). A similar form does also occur in adults. Exercise-induced asthma can be diagnosed in children or adults and is characterized by relatively mild, intermittent symptoms. Biologically there is mast cell activation, T2 high and it tends to respond to leukotriene antagonists, beta agonists and IL-9 agonists (94).

#### Molecular

More recently molecular phenotyping has also been attempted. In one study on mild to moderate asthma patients upregulated genes were identified in epithelial brushings to molecularly classify those with T2-high and T2-low asthma (90). Those with T2-low asthma were found to have similar gene-expression patterns to the control group (90). Those with T2-high asthma on genetic profile were found to have increased IL-13, IL-5, eosinophils, and mast cells as well as more atopy (95).

Molecular phenotyping has potential implications for treatment with those with T2-high asthma based on genetic profile having a response to inhaled corticosteroids and those with T2-low disease having no response (90, 95).

### Adult Phenotypes of Asthma

#### Late Onset Eosinophilic

Characterized by both clinical and biologic features of later onset, predominately female, and elevated sputum and serum eosinophils. Late-onset eosinophilic asthma is defined clinically by adult-onset, severe disease and is associated with sinusitis and less allergic sensitization compared to early onset disease. Biologically patients have increased IL-5 and IL-13 in the airways and elevated eosinophils in the sputum and serum (96–98). No cut off for sputum and serum eosinophils have been universally agreed upon however it is generally accepted that a sputum eosinophil count of >2% or a serum eosinophil count of >300 cells/uL (or in some cases >150 cells/uL) indicates eosinophilic asthma (99–101). Despite a high prevalence of positive skin prick tests this form of asthma appears to be less allergic but is often associated with sinusitis, nasal polyps, and aspirin exacerbated respiratory disease (96). A family history of asthma is seen less frequently than those with early onset asthma (96). This type of asthma can be relatively steroid resistant but biologic therapies targeting T2 pathways have been shown to be highly effective in this group of patients (102–105).

#### Obesity-Related Asthma

Obesity-related asthma is not well-understood. It is unclear whether it is a comorbidity common in asthma that confers greater likelihood of breathing pattern disorder, gastroesophageal reflux and deconditioning or whether it is the driver for a proinflammatory state that results in asthma (106–109). Higher body mass index (BMI) is associated with increased levels of TNFa, IL-6, and leptins and less eosinophils, FeNO and corticosteroid responsiveness (108, 109). Clinically there appears to be a group of older non-allergic, obese females who have significant symptoms but minimal healthcare utilization (76, 77). The interaction between BMI and eosinophils is more complex, with those who have early onset T2-high asthma having a correlation between duration of asthma and BMI, lower activity levels, and corticosteroid exposure that does not appear to exist in those with T2-low disease (110). Bariatric surgery has been shown to improve outcomes in asthma patients with late onset, non-allergic asthma but not in those with allergic disease (111).

#### Neutrophilic Asthma

Neutrophilic asthma is poorly defined and there is no consensus about the characterization of this entity. Adding to the confusion is the fact that corticosteroid treatment commonly suppresses eosinophils and causes neutrophilia making the assessment of corticosteroid-dependant patients difficult (112–114). The clinical phenotype remains controversial and inconsistent but has been suggested to be that of adult-onset, severely obstructed with only partial reversibility and a high healthcare utilization (77, 78). Smoking may also play a role. Furthermore, neutrophilic asthma can occur in those with elevated eosinophils conferring a particularly severe clinical phenotype (115). These patients tend to be less corticosteroid responsive and other treatment strategies have been tried including use of macrolide antibiotics (116). Although IL-17 has been suggested as a potential therapeutic target in neutrophilic asthma a biologic targeted at this interleukin did not result in improvement in asthma control and so far this area remains disappointing (117, 118). The initial disappointment with targeted therapies such as the anti-IL-17 and anti-TNFa agents has been tempered by positive preliminary results with a newer biologic agent targeting TSLP that has showed promise in Phase 2 studies in a non-eosinophilic group (119, 120).

#### Aspirin-Associated Asthma

Aspirin-associated asthma, a subset of aspirin exacerbated respiratory disease (AERD) has been described for many years (85). It tends to occur in adulthood at an average age of 34 years and is more common amongst females (86). This is a subset of late-onset eosinophilic asthma and is associated with sinusitis, nasal polyps and sensitivity to cyclooxygenase-1 inhibitors including aspirin (86). Biologically it is characterized by upregulation of the cysteinyl leukotriene pathway and elevated eosinophils (96). Molecularly genes related to the leukotriene pathway have been implicated and periostin, a biomarker of IL-13 activity, has been found in nasal polyps present in patients with AERD (97, 121). These patients are often relatively corticosteroid resistant, requiring high doses for control, but can be responsive to leukotriene antagonists (122, 123). More recently biologic therapies that target T2 pathways including IL-4, IL-13, and IL-5 have been shown to be effective in patients with asthma and nasal polyps (124–126).

#### Allergic Bronchopulmonary Aspergillosis

Allergic bronchopulmonary aspergillosis (ABPA) was first described in the 1950s and is caused by allergic sensitization to fungal colonization of the lower airways with *Aspergillus fumigatus* (127, 128). It occurs in 1–2% of asthma patients, although has been detected in up to 13% of the population in asthma clinics, predominately adults, and causes asthma exacerbations, deterioration of pulmonary function, mucous plugging, central bronchiectasis, and transient pulmonary infiltrates with characteristic biologic features including elevated total and Aspergillus-specific IgE as well as peripheral eosinophilia (88, 128, 129). The diagnosis is based on presence of asthma, proximal bronchiectasis, sensitization to Aspergillus, and an elevated total IgE (129). It is important to diagnose ABPA because of the progressive nature of the bronchiectasis in the absence of treatment (129). The mainstay of treatment for ABPA is systemic corticosteroids and, in some cases, antifungal agents (88).

#### Link Between Early Transient Wheeze and COPD

There is a link between childhood asthma and COPD (130). Both childhood asthma and childhood wheezy bronchitis have been associated with an increased risk of COPD in adults (131). Severe childhood asthma has been shown to confer a 32 times higher risk of COPD in adults despite the fact that just under half of those diagnosed with COPD in this cohort had never smoked (132). Early transient wheeze has been thought of as a benign condition but did significantly increase risk of the presence of COPD in adulthood with long term follow up (131).

Asthma-smoking associations have been described in both early and late onset asthma (133). Smoking remains a key risk factor for airflow obstruction in normal and asthmatic individuals, however the risk appears to be greatest in those with late onset disease (74).

### Natural History

The natural history of asthma in adults is different to that of asthma in children with less remission of adult-onset asthma than that occurring in childhood.

Many adults with asthma have childhood-onset disease that has persisted and there have been many risk factors that predict the persistence of asthma into adulthood including the severity of childhood disease, the presence of bronchial hyperresponsiveness, atopy, exposure to allergens and a parental history of asthma (134–136). In longitudinal studies the amount of wheeze in early adolescence has been shown to predict the severity in later life. In an Australian-based cohort of second graders with wheezing of various severities followed up to age 50 there was remission of asthma in 64% of those with mild wheezy bronchitis or wheezy bronchitis, 47% of those with persistent asthma and only 15% of those with severe asthma (137). In this group risk factors for persistence of asthma at age 50 were severe childhood asthma, childhood allergic rhinitis, and female sex (137). In another longitudinal cohort 73% with few symptoms at 14 years had little or no asthma 14 years later whereas 68% of those with frequent wheeze at 14 years still had asthma 14 years later (138). Most who had frequent wheeze at 21 still had wheeze at 28 years and of those with infrequent wheeze at 21 years 44% had worsened at 28 years (138). These findings have been replicated in more recent studies—three quarters of children with childhood asthma will outgrow the disease by middle age although overall our understanding of the natural history of childhood asthma remains poorly understood (139).

Adult onset asthma has many different forms and the risk factors appear to be different to that of childhood-onset disease. Compared to childhood asthma, major associations with adult-onset disease are female sex, current smoking, and low socio-economic status but not atopy or a family history of asthma (74). Other risk factors include; clinical—historical symptoms of wheeze, rhinitis, chronic cough; physiological—lower lung function, bronchial hyperresponsiveness, lower height; comorbidities—higher BMI, nocturnal gastroesophageal reflux disease, habitual snoring, IgE reactivity to Timothy grass; and lifestyle—low physical activity amongst men (75, 107, 140–144). The evidence for smoking as a risk factor is mixed with two large cohort studies from Australia and Sweden showing that this is a risk factor for adult-onset asthma and other studies showing that it doesn't appear to be (74, 75, 141, 144).

In a large cohort with severe asthma with onset between 14 and 55 who were followed for 10 years 83% had less severe asthma at 10 years (145). Risk factors for the persistent presence of severe asthma was low socioeconomic status, high comorbidity burden and high adherence to medications in the first year after diagnosis (145). In this cohort sex and other risk factors important in childhood asthma were not associated with continued presence of severe asthma (145).

The sex differences in asthma amongst different age groups are interesting. Amongst children males are more commonly affected by asthma than females and male sex is a risk factor for developing asthma (39). Around the time of puberty this risk becomes equal and after puberty the risk in females is greater than that amongst males, an observation partially but not fully explained by women's smaller airway caliber in adults (39). There is emerging evidence of the association between female hormonal changes and asthma that may partially explain the female predominance of asthma after puberty (146, 147).

Some factors that don't appear to be risk factors for adult onset asthma include level of education, atopy (either baseline or newly positive skin prick tests), occupational exposures or maternal asthma (141, 143, 144).

Despite the differences in risk factors for adult-onset asthma compared to childhood onset disease the prevalence of asthma amongst adults was shown to be increasing in the second half of the twentieth century similar to that of childhood asthma (73, 148).

#### Young Adults

In those who develop asthma as young adults the natural history appears to be more similar to that of childhood asthma with atopy an important a risk factor and more remissions (149). The 23 year follow up of one study in the US examined asthma incidence in participants 23 years after college, finding that the cumulative prevalence of asthma increases with age and that three-quarters of those who had asthma at the first visit were in remission or had improved symptoms at 40 years (149). Only a small number who had asthma at the baseline visit were worse (149).

#### Middle Age

Many patients are diagnosed with asthma in middle age and this disproportionately affects women. In fact for women most asthma occurring in middle age is adult-onset asthma and by 40 years more than half of asthma in women is adult onset (73). The proportion of adult onset asthma was even higher in women who were obese, non-atopic, ever smokers, white, and lower socio-economic status (73, 74). In contrast for men by 50 years only one third of asthma is adult-onset asthma (73). Therefore it is clear that adult-onset asthma is more common in women (73). Overall those with adult onset asthma are more likely to experience symptoms including wheeze, new rhinitis, snoring, and weight gain (143). Those with adult onset disease are also more likely to have a decline in lung function than those with childhood onset disease (143).

#### Older Adults

For those who first experience asthma in their senior year atopy does not appear to be a risk factor (149). Older adults who develop asthma have a similar incidence as younger people of ~100 per 100,000 (150). However, disease severity is worse amongst older adults developing asthma compared to younger people and is also more progressive with poorer lung function and more fixed airflow obstruction (151). This poorer lung function and more fixed airflow obstruction is thought to be largely the result of the more common presence of comorbid lung diseases such as bronchiectasis, pulmonary fibrosis, and COPD (151). Older adults with asthma are less likely to experience remission than their younger counterparts (151).

#### Remissions

In adults with asthma remissions have been found to be uncommon after the second decade of life and particularly uncommon in those between 30 and 60 years old (79). In adult-onset, non-allergic asthma remission is especially unusual occurring in only 20% of patients (152). Risk factors for those not achieving remission were severe symptoms, impaired lung function, and a comorbid diagnosis of chronic bronchitis or emphysema (79). Amongst adults with asthma who do remit relapses have been found to be common and increase until the age of 70 years particularly if there was persistent symptoms despite remission (79, 153).

#### Lung Function

Lung function has been found to decline in some but not all adult-onset asthma patients (154–156). In those with persistent asthma there is generally a decline in lung function that eventually leads to a restrictive pattern of spirometry (157). Adult lung function is influenced by initial FEV1 and sex, with a larger decline observed amongst females, but not initial asthma severity or allergic sensitization (136). Lower lung function has also been found to be related to presence of bronchial hyperresponsiveness (136).

In aspirin-exacerbated asthma a steady progress in severity has been found (86). Rhinitis generally appears around the age of 30 years followed by asthma, aspirin sensitivity, then nasal polyps (158). Women generally have more severe disease with earlier onset, one third of patients are atopic and they tend to have an earlier onset, and lower FEV1 (159).

The natural history of ABPA is variable with five stages identified including acute, active; remission; recurrent; chronic; and severe, end-stage disease (160). Early diagnosis and treatment is associated with a reduced likelihood of progressive disease (146, 160). Serum IgE level is often used to track progress of disease and help to identify remission and relapse of ABPA (160). Overall those with ABPA have been observed to have a progressive decline in lung function although there are those who undergo complete remission (88).

#### Mortality

Thankfully mortality in asthma remain uncommon (71). However mortality rates are not declining and in fact have increased in some developed countries (71, 161). Asthma deaths are more common amongst adolescents and young adults, those of low socioeconomic status, black race, those with substance abuse, and are uncommon in young children and older adults (162). Other risk factors for death include previous near-fatal asthma, hospitalization or emergency department visit for asthma in the past year, current or recent oral corticosteroid use, non-adherence with inhaled corticosteroids, a history of psychiatric disease, lack of a written asthma action plan and the presence of comorbid food allergy (161). Overall the average life expectancy for those with asthma is not reduced compared to the general population (163). Elderly patients die more frequently from respiratory diseases and are more at risk of complications of medications (150).

### Contrasts

It is difficult to accurately compare and contrast childhood and adult-onset asthma due to existing gaps in the literature and we acknowledge this limitation. Additionally because some findings are reported more in adults, this does not necessarily mean they are more prevalent, but rather a possible manifestation of publication bias. Nevertheless, we have provided a reflection of the similarities and differences based on the currently available literature (Tables 1, 2). Adult onset disease differed from pediatric onset disease in regard to increased prevalence in women, non-atopic individuals, and obese patients (73). Adult onset disease is associated with more respiratory symptoms and asthma medication use despite higher prebronchodilator FEV1/FVC (73). There is less quiescent disease in adult onset asthma and it appears to be less stable than childhood-onset disease with more relapses and less remissions (73).

**Table 2:** Asthma presentations across the lifecourse.

**Age**	**Presentation/Phenotype**	**Description**
Child	Asthma diagnosis	Physician's diagnosis of asthma at least once per lifetime or recurrent diagnoses of spastic, obstructive, or asthmatic bronchitis as reported by the parents at age 6 years
	Frequent wheeze	Wheeze on a monthly basis for at least 1 year between age 1 and 6 years
	Unremitting wheeze	Having symptoms between wheezing episodes or having wheeze without a cold at least once between age 1 and 6 years
	Recurrent unremitting wheeze	Having *s*ymptoms between wheezing episodes or wheeze without a cold for 2 or more years between age 1 and 6 years
	Multi-Trigger wheeze	Having at least 2 common asthma triggers leading to wheeze between ages 3 and 6 years
	Episodic wheeze	Wheezing episodes associated only with viral upper respiratory infection between age 1 and 6 years.
	Severe asthma Difficult to control asthma Therapy resistant asthma	Asthma which is poorly controlled based on frequent symptoms and significant morbidity Often due to incorrect diagnosis, poor adherence to therapy, incorrect inhaler/spacer technique, uncontrolled comorbidities (allergic rhinitis) Poorly controlled asthma after ruling out “Difficult to control asthma”
	Eosinophilic predominant asthma	Allergic asthma
	Neutrophilic predominant asthma	Non-allergic asthma
	Recurrent croup	Repeated episodes of croup
	Middle lobe syndrome	Repeated episodes of middle lobe infiltrate or atelectasis
	Recurrent pneumonia	Repeated episodes of lung infection
Adult	Late onset eosinophilic asthma	Later onset, predominately female, and elevated sputum and serum eosinophils, associated with sinusitis
	Obesity related asthma	Associated with increased levels of TNFa, IL-6, leptins, less eosinophils, FeNO, and corticosteroid responsiveness
	Neutrophilic asthma	Difficult to characterize, often severely obstructed with only partial reversibility and a high healthcare utilization
	Aspirin- associated asthma	Subset of late-onset eosinophilic asthma, associated with sinusitis, nasal polyps, and sensitivity to cyclooxygenase-1 inhibitors
	Allergic bronchopulmonary aspergillosis	Lower airway allergic sensitization to *Aspergillus fumigatus* causing asthma exacerbations, pulmonary function deterioration, mucous plugging, central bronchiectasis, and transient pulmonary infiltrate

Severe asthma in children is distinct from severe asthma in adults and approaches to severe asthma in adults should not be extrapolated to children. In children the factors associated with severity have been found to be asthma duration, medication use and lung function rather than Type 2 inflammatory markers such as increased IgE and elevated FENO that are markers of severity in adult-onset disease (93).

## Author Contributions

MT and ED made substantial contributions to: The conception or design of the work; or the acquisition, analysis, or interpretation of data for the work; Drafting the work or revising it critically for important intellectual content; Provide approval for publication of the content; Agree to be accountable for all aspects of the work in ensuring that questions related to the accuracy or integrity of any part of the work are appropriately investigated and resolved.

### Conflict of Interest Statement

The authors declare that the research was conducted in the absence of any commercial or financial relationships that could be construed as a potential conflict of interest.
